# Using dynamic clamp to quantify pathological changes in the excitability of primary somatosensory neurons

**DOI:** 10.1113/JP275580

**Published:** 2018-05-10

**Authors:** Petri Takkala, Steven A. Prescott

**Affiliations:** ^1^ Neurosciences and Mental Health The Hospital for Sick Children Toronto Ontario Canada M5G 0A4; ^2^ Institute of Medical Science University of Toronto Toronto Ontario Canada M5S 1A8; ^3^ Department of Physiology and Institute of Biomaterials and Biomedical Engineering University of Toronto Ontario Canada M5S 1A8

**Keywords:** Dynamic clamp, Dorsal Root Ganglion, Pain, Spiking pattern, Hyperexcitability, Inflammation, Neuronal excitability, Neuropathic pain

## Abstract

**Key points:**

- Primary somatosensory neurons normally respond to somatic depolarization with transient spiking but can switch to repetitive spiking under pathological conditions. This switch in spiking pattern reflects a qualitative change in spike initiation dynamics and contributes to the hyperexcitability associated with chronic pain.
- Neurons can be converted to repetitive spiking by adding a virtual conductance using dynamic clamp. By titrating the conductance to determine how much must be added to cause repetitive spiking, we found that small cells are more susceptible to switching (i.e. required less added conductance) than medium–large cells.
- By measuring how much less conductance is required to cause repetitive spiking when dynamic clamp was combined with other pathomimetic manipulations (e.g. application of inflammatory mediators), we measured how much each manipulation facilitated repetitive spiking.
- Our results suggest that many pathological factors facilitate repetitive spiking but that the switch to repetitive spiking requires the cumulative effect of many co‐occurring factors.

**Abstract:**

Primary somatosensory neurons become hyperexcitable in many chronic pain conditions. Hyperexcitability can include a switch from transient to repetitive spiking during sustained somatic depolarization. This switch results from diverse pathological processes that impact ion channel expression or function. Because multiple pathological processes co‐occur, isolating how much each contributes to switching the spiking pattern is difficult. Our approach to this challenge involves adding a virtual sodium conductance via dynamic clamp. The magnitude of that conductance was titrated to determine the minimum required to enable rheobasic stimulation to evoke repetitive spiking. The minimum required conductance, termed ${\bar{g}}_{\mathrm{Na}}^{*}$, was re‐measured before and during manipulations designed to model various pathological processes *in vitro*. The reduction in ${\bar{g}}_{\mathrm{Na}}^{*}$ caused by each pathomimetic manipulation reflects how much the modelled process contributes to switching the spiking pattern. We found that elevating extracellular potassium or applying inflammatory mediators reduced ${\bar{g}}_{\mathrm{Na}}^{*}$ whereas direct hyperpolarization had no effect. Inflammatory mediators reduced ${\bar{g}}_{\mathrm{Na}}^{*}$ more in medium–large (>30 μm diameter) neurons than in small (⩽30 μm diameter) neurons, but had equivalent effects in cutaneous and muscle afferents. The repetitive spiking induced by dynamic clamp was also found to differ between small and medium–large neurons, thus revealing latent differences in adaptation. Our study demonstrates a novel way to determine to what extent individual pathological factors facilitate repetitive spiking. Our results suggest that most factors facilitate but do not cause repetitive spiking on their own, and, therefore, that a switch to repetitive spiking results from the cumulative effect of many co‐occurring factors.

## Key points

- Primary somatosensory neurons normally respond to somatic depolarization with transient spiking but can switch to repetitive spiking under pathological conditions. This switch in spiking pattern reflects a qualitative change in spike initiation dynamics and contributes to the hyperexcitability associated with chronic pain.
- Neurons can be converted to repetitive spiking by adding a virtual conductance using dynamic clamp. By titrating the conductance to determine how much must be added to cause repetitive spiking, we found that small cells are more susceptible to switching (i.e. required less added conductance) than medium–large cells.
- By measuring how much less conductance is required to cause repetitive spiking when dynamic clamp was combined with other pathomimetic manipulations (e.g. application of inflammatory mediators), we measured how much each manipulation facilitated repetitive spiking.
- Our results suggest that many pathological factors facilitate repetitive spiking but that the switch to repetitive spiking requires the cumulative effect of many co‐occurring factors.

## Introduction

Neurons use action potentials, or spikes, to transmit information. Spike generation depends on neuronal excitability. If excitability is misregulated, neurons may produce too many or too few spikes in response to input, thus compromising the encoding of that input. Primary afferent neurons, which relay somatosensory information from the periphery (e.g. skin and muscle) to the central nervous system, can become hyperexcitable in chronic pain conditions (Devor, 2005). In fact, primary afferent hyperexcitability is necessary and sufficient to explain the hypersensitivity and spontaneous pain associated with many chronic pain conditions (Gracely *et al*. 1992; Gold & Gebhart, 2010; Shankarappa *et al*. 2012; Vaso *et al*. 2014). One of the more dramatic manifestations of cellular hyperexcitability is a switch in spiking pattern during sustained somatic depolarization (Xing *et al*. 2001; Liu *et al*. 2002; Fan *et al*. 2011; Ratté *et al*. 2014). The switch from transient to repetitive spiking reflects a qualitative change in spike initiation dynamics (Rho & Prescott, 2012). This switch not only dramatically amplifies evoked spiking, it also enables spontaneous spiking insofar as spontaneous spiking requires a depolarizing shift in resting membrane potential *and* the capacity to spike repetitively when depolarized. Understanding the transition from transient to repetitive spiking is thus an important goal.

This pathological switch in spiking pattern can arise through changes in many different ion channels (Rho & Prescott, 2012; Ratté *et al*. 2014). Equivalent changes in excitability arising through disparate molecular changes is an example of degeneracy (Edelman & Gally, 2001) and has critical implications for understanding and treating chronic pain (Ratté & Prescott, 2016). Indeed, primary somatosensory neurons express diverse ion channels (Dib‐Hajj *et al*. 2010; Du & Gamper, 2013; Waxman & Zamponi, 2014). Inflammation and nerve injury impact those channels via transcriptional, translational and post‐translational changes, and can also impact cytokine receptors whose up‐ or downregulation modulates effects of inflammation on ion channel function, amongst other changes (Woolf & Costigan, 1999; Gold & Gebhart, 2010; LaCroix‐Fralish *et al*. 2011; Chahine & O'Leary, 2014; Waxman & Zamponi, 2014; Laedermann *et al*. 2015; Ji *et al*. 2016).

Because inflammation and nerve injury trigger many molecular changes that influence excitability, ascribing a shift in excitability to a particular molecular change requires that one account for co‐occurring molecular changes. An alternative for establishing such links is to experimentally reproduce a molecular change in isolation, for example, by blocking a specific ion channel pharmacologically or enhancing it via dynamic clamp, an electrophysiological technique whereby virtual conductances can be introduced into real neurons (Robinson & Kawai, 1993; Sharp *et al*. 1993). Using dynamic clamp, one can titrate the virtual conductance to determine how much of it must be added to a neuron to make it spike repetitively (Ratté *et al*. 2014). This sort of testing not only provides valuable insight into how the virtual conductance affects excitability, it can also be used to investigate the susceptibility of different neurons to switch their spiking pattern and whether that susceptibility is affected by changes in native ion channels (see below). In other words, one can (i) study the effects of the added conductance or (ii) use the effects of the added conductance to study other factors that affect spiking pattern. We focus here on the latter.

Excitability can be measured in many ways. Each metric should ideally provide independent information, but this is not always true in practice. For instance, if inflammation depolarizes a neuron, the current required to evoke spiking – rheobase – will be reduced. For rheobase to provide information independent from voltage measurements, the confounding change in voltage must be controlled when measuring rheobase. With that in mind, we controlled for changes in resting membrane potential and rheobase when measuring the minimum virtual sodium conductance required to cause repetitive spiking, henceforth referred to as ${\bar{g}}_{\mathrm{Na}}^{*}$. Changes in ${\bar{g}}_{\mathrm{Na}}^{*}$ were observed despite those controls (see Results), consistent with the minimum virtual conductance required to switch the spiking pattern (i.e. ${\bar{g}}_{\mathrm{Na}}^{*}$) being fundamentally different from the current required to evoke spiking (i.e. rheobase). In the present study, we have monitored (and reported) changes in resting membrane potential, rheobase and other membrane properties, but our goal was to quantify how much specific pathological conditions facilitate the switch to repetitive spiking.

We demonstrate here how dynamic clamp can be combined with manipulations designed to model specific pathological conditions *in vitro* in order to quantify the effect of those conditions on spiking pattern (see Methods). Our approach involves asking *how much less* virtual conductance must be added to a neuron to switch its spiking pattern when the virtual conductance is added in conjunction with a pathomimetic manipulation; in other words, how much a certain condition (or factor) facilitates repetitive spiking is revealed by how much it reduces ${\bar{g}}_{\mathrm{Na}}^{*}$. Using this approach, we quantified how much elevated extracellular potassium and inflammatory mediators – two factors associated with chronic pain conditions (Marchand *et al*. 2005; Devor, 2006; Gold & Gebhart, 2010; Amaya *et al*. 2013) – reduced ${\bar{g}}_{\mathrm{Na}}^{*}$ and whether the reduction varied between different types of primary somatosensory neurons. Whereas both of those manipulations reduced ${\bar{g}}_{\mathrm{Na}}^{*}$, direct neuronal hyperpolarization did not affect ${\bar{g}}_{\mathrm{Na}}^{*}$ despite increasing rheobase. Thus, ${\bar{g}}_{\mathrm{Na}}^{*}$ does not duplicate traditional excitability metrics but, instead, reflects how far from its tipping point a neuron normally operates. Our results suggest that individual pathological factors facilitate but do not cause a switch in spiking pattern (i.e. they push the neuron toward but not across its tipping point), which suggests that a switch to repetitive spiking typically results from the cumulative effect of multiple co‐occurring factors rather than because of any single factor.

## Methods

### Ethical approval

All procedures were approved by the Animal Care Committee at The Hospital for Sick Children and were conducted in accordance with guidelines from the Canadian Council on Animal Care. The authors understand the ethical principles under which *The Journal of Physiology* operates and ensure full compliance with the guidelines for the ethical use of animals, as described by Grundy (2015).

### Animals

Adult (150–250 g) male Sprague–Dawley rats (Charles River, Sherbrooke, Canada) were used for all experiments. Rats were housed under standard conditions in a 12 h light/dark cycle and were provided food and water *ad libitum*.

### Retrograde labelling of cutaneous or muscle afferent neurons

Retrograde labelling was used to target *in vitro* those neurons that innervate a particular tissue *in vivo*. To identify neurons innervating the glabrous skin of the hind paw, the fluorescent dye di‐alkyl indocarbocyanine (DiI; Sigma‐Aldrich, Oakville, ON, Canada) was injected into the glabrous skin. Details of DiI labelling have been previously described (Gold & Traub, 2004). Briefly, DiI (3.4 mg) was prepared in a solution of 20 μl dimethyl sulphoxide (DMSO; Amresco, Mississauga, ON, Canada), which was diluted to a 1:10 solution in 0.9% sterile saline. Under isoflurane anaesthesia (4% induction, 2.5% maintenance), 10 μl of DiI solution was injected intradermally into five sites (2 μl per site) on the plantar surface of the hind paw. Alternatively, to label afferents innervating muscle, 10 μl of DiI solution was injected into five sites of the gastrocnemius muscle. To exclude unintended labelling of cutaneous afferents along the needle track, 10 μl of Fast Blue (1% in sterile saline) was injected intradermally around the intramuscular injection site. Animals were monitored for signs of inflammation or behavioural abnormalities for 10–20 days post‐injection, but none required analgesia or other interventions. After 10–20 days, DiI‐labelled somata were identified in acute dorsal root ganglion (DRG) cultures by epifluorescent imaging of DiI using Zeiss filter set 43. Absence of Fast Blue labelling was confirmed for muscle afferents using Zeiss filter set 02.

### Culture preparation

Each rat was anaesthetized with isoflurane (4% induction, 2.5% maintenance) and a laminectomy was performed to expose the lumbar DRG. The L_4_ and L_5_ DRG were surgically excised and the rat was then killed by cervical dislocation under deep anaesthesia. Excised DRG were desheathed in chilled culture medium consisting of: 89% minimum essential medium (MEM), 10% fetal bovine serum (FBS), 100 units ml^−1^ of penicillin and 100 μg ml^−1^ streptomycin, and supplemented with 1% MEM vitamin solution, all from Gibco Life Technologies (Waltham, MA, USA) and henceforth referred to as MEM‐FBS. Desheathed DRG were enzymatically treated for 45 min in culture media composed of: 89% MEM, 370 units ml^−1^ penicillin and 370 μg ml^−1^ streptomycin, 1% MEM vitamin solution, and 1.2 mg ml^−1^ collagenase Type 4 (Worthington Biochemical, Lakewood, NJ, USA), maintained at 37°C and continuously bubbled with carbogen (95% O_2_–5% CO_2_). DRG were mechanically dissociated by trituration with a fire‐polished Pasteur pipette, and further enzymatically treated for 5 min in Ca^2+^‐ and Mg^2+^‐free Hanks’ balanced salt solution (HBSS; Gibco Life Technologies), containing 2.5 mg ml^−1^ trypsin (Worthington Biochemical) and 0.02% sterile ethylenediaminetetraacetic acid (EDTA; Sigma‐Aldrich). Trypsin activity was subsequently inhibited by the addition of MEM‐FBS supplemented with 0.625 mg ml^−1^ MgSO_4_. Dissociated cells in MEM‐FBS were plated onto glass coverslips coated with poly‐d‐lysine (Fisher Scientific, Waltham, MA, USA), and incubated at 37°C, 5% CO_2_ and 90% humidity for 2 h. After incubation, coverslips were stored for 12–24 h at room temperature in media composed of HEPES‐buffered Leibovitz's L‐15 medium containing glutamine (Gibco Life Technologies), 10% FBS, 100 units ml^−1^ of penicillin and 100 μg ml^−1^ streptomycin, and 5 mm d‐glucose (Caledon Laboratories, Georgetown, ON, Canada).

### Recordings and data acquisition

For patch clamp recordings, coverslips were transferred to a recording chamber perfused at 2 ml min^−1^ with artificial cerebral spinal fluid (ACSF) consisting of (in mm): 126 NaCl, 2.5 KCl, 2 CaCl_2_, 2 MgCl_2_, 10 d‐glucose, 26 NaHCO_3_, and 1.25 NaH_2_PO_4_; pH = 7.4, osmolality = 300 mosmol kg^−1^, and bubbled continuously with carbogen (95% O_2_–5% CO_2_). To cause a depolarizing shift in equilibrium potential for potassium (*E*_K_) in certain experiments, KCl was increased to 5.5 mm, and NaCl was decreased to 123 mm to maintain solution osmolarity. To reproduce the effects of acute inflammation, the inflammatory mediators histamine, bradykinin and prostaglandin E_2_ (PGE_2_) were added to ACSF to a final concentration of 10 μm each (Ma *et al*. 2006). All salts were obtained from Caledon Laboratories and inflammatory mediators were obtained from Sigma‐Aldrich.

The somatic diameter of DiI‐labelled neurons was measured using an ocular graticule under gradient contrast optics using a AxioExaminer microscope (Zeiss, North York, ON, Canada) fitted with a 40× /0.75 NA water immersion objective. DiI‐labelled DRG neurons were patched using a 5 MΩ micropipette containing an internal solution composed of (in mm): 125 KMeSO_4_, 5 KCl, 10 HEPES, 2 MgCl_2_, 4 adenosine triphosphate (ATP), 0.4 guanosine triphosphate (GTP), and included 0.1% Lucifer Yellow; pH adjusted to 7.2 with KOH, osmolality = 290 mosmol kg^−1^. Whole‐cell patch clamp recordings were acquired using an Axopatch 200B amplifier (Molecular Devices, Sunnyvale, CA, USA) with >70% series resistance compensation. Data were low‐pass filtered at 2 kHz and digitized at 20 kHz using a CED 1401 computer interface and CED Signal 5 software (Cambridge Electronic Design, Cambridge, UK). All recordings were made at room temperature (22–24°C) unless otherwise noted.

Throughout each protocol, current injection was continuously applied to adjust the membrane potential (*V*_m_) to –65 mV except when measuring the resting *V*_m_. Stimulating current (*I*_stim_) was applied as 0.5 s‐long steps to measure the membrane time constant (τ_m_), input resistance (*R*_in_), and rheobase of each neuron. A small (50 pA) hyperpolarizing current step was used to determine τ_m_ from a single exponential curve fit, and to calculate *R*_in_ from the change in membrane potential (*R*_in_ = Δ*V*_m_/*I*_stim_). Rheobase was determined as the minimum depolarizing current required to elicit an action potential. The membrane capacitance (*C*_m_) was calculated from τ_m_ and *R*_in_ (*C*_m_ = τ_m_/*R*_in_). All values of voltage were corrected for a –9 mV liquid junction potential.

Two second‐long current steps were used to probe spiking pattern. Repetitive spiking was defined as a response of *consistently* more than one spike across repeated trials at a given *I*_stim_ intensity; in practice, repetitive spiking was identified as more than three spikes on a single trial because, according to preliminary testing, this always translated into more than one spike during repeated testing with the same *I*_stim_ intensity (but repeated testing was not feasible when covarying *I*_stim_ and ${\bar{g}}_{\mathrm{Na}}$ under multiple conditions). Transient spiking typically comprised a single spike at stimulus onset but, when a neuron was near its tipping point, sometimes included an additional one or two *inconsistent* spikes (see above). Spiking pattern can be determined across a range of stimulus intensities but the transition from transient to repetitive spiking was always determined at rheobase, and subtypes of repetitive spiking (namely phasic or tonic) were also determined at rheobase. Requiring consistently more than one spike to designate the spiking pattern as “repetitive” ensured that we did not underestimate ${\bar{g}}_{\mathrm{Na}}^{*}$ (see below). When testing with *I*_stim_ steps, transient spiking reflects spike initiation through a quasi‐separatrix crossing whereas repetitive spiking reflects spike initiation through a Hopf bifurcation according non‐linear dynamical analysis (Prescott *et al*. 2008; Rho & Prescott, 2012). Notably, a “transient spiking” neuron may spike repetitively during *I*_stim_ ramps (Ratté *et al*. 2015), which illustrates the importance of the stimulus protocol. *I*_stim_ current steps are commonly employed in primary afferent studies and can distinguish between spike initiation mechanisms (see above), and thus satisfy our needs.

### Inserting virtual Na_V_1.3‐like conductance using dynamic clamp

A virtual Na^+^ conductance (*g*_Na_) with activation kinetics based on a Hodgkin–Huxley model was added to patch clamp recordings using a dynamic clamp protocol. Details of the dynamic clamp protocol have been described previously (Ratté *et al*. 2014). Briefly, *g*_Na_ was modelled according to:

I Na =g¯ Na mV−E Na ,

$$\frac{dm}{dt}=\alpha \left(1-m\right)-\beta m,$$

$$\alpha =k_{\alpha }\frac{\left(\frac{V-V_{\alpha }}{s_{\alpha }}\right)}{e^{\left(\frac{V-V_{\alpha }}{s_{\alpha }}\right)}-1},$$

$$\beta =k_{\beta }e^{\left(\frac{V-V_{\beta }}{s_{\beta }}\right)}.$$

The maximal conductance (${\bar{g}}_{\mathrm{Na}})\;$was adjusted as described below. All other parameters were set as follows: *E*_Na_ = 50 mV, $k_{\alpha ,\beta }=$ 1.5 m s^−1^, $V_{\alpha ,\beta }$ = –15 mV, $s_{\alpha ,\beta }$ = –17 mV. The virtual conductance resembles but was not strictly designed to model Na_V_1.3 channels; instead, conductance parameters were identified through an agnostic parameter search in simulations focused on changes in spike initiation mechanism (Rho & Prescott, 2012; Ratté *et al*. 2014).

**Figure 1:**
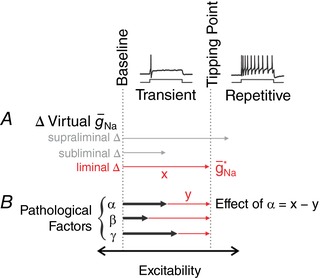
Measuring excitability changes that affect spiking pattern Most primary somatosensory neurons respond to sustained somatic current injection (*I*_stim_) with transient spiking but some switch to repetitive spiking under pathological conditions. That switch occurs at a “tipping point” where the non‐linear mechanism responsible for spike initiation undergoes a qualitative change. *A*, the distance to tipping point can be measured by titrating how much virtual Na_V_1.3‐like conductance (see Methods) is required to switch the spiking pattern. We refer to the liminal change (red arrow) as ${\bar{g}}_{\mathrm{Na}}^{*}$. Notably, Na_V_1.3 channels are one of many different ion channels whose up‐ or downregulation impacts the spike initiation mechanism; the virtual Na_V_1.3‐like conductance is used here purely as a tool to help infer the impact of other factors on spiking. *B*, pathological factors affecting excitability (represented here as black arrows labelled α, β, γ) may *facilitate* repetitive spiking without *causing* repetitive spiking (i.e. shift a neuron toward but not across its tipping point). We reasoned that the degree to which each factor facilitated repetitive spiking could be measured as the difference in ${\bar{g}}_{\mathrm{Na}}^{*}$ before (*x*) and during (*y*) each pathomimetic manipulation.

To determine the minimum ${\bar{g}}_{\mathrm{Na}}$ required to change the spiking pattern from transient to repetitive spiking, represented as ${\bar{g}}_{\mathrm{Na}}^{*}$, *I*_stim_ steps were varied to within 50 pA of rheobase and ${\bar{g}}_{\mathrm{Na}}$ was systematically varied until repetitive spiking was observed in response to *I*_stim_. To compare ${\bar{g}}_{\mathrm{Na}}^{*}$ across neurons of different sizes, values of ${\bar{g}}_{\mathrm{Na}}$ were converted to conductance density by dividing by membrane capacitance (${\bar{g}}_{\mathrm{Na}}^{*}$/*C*_m_). Measurements of ${\bar{g}}_{\mathrm{Na}}^{*}$ and other membrane properties were conducted in standard ACSF and again during each manipulation (elevated [K^+^]_o_ or added inflammatory mediators). To measure how much a pathological factor facilitated repetitive spiking, we compared ${\bar{g}}_{\mathrm{Na}}^{*}$ in the same neuron before and during each pathomimetic manipulation (Fig. 1).

### Statistical analysis

Data were analysed using SigmaPlot 11.0 software (Systat Software Inc., San Jose, CA, USA). Proportions were compared using a Yate's corrected χ^2^ test, a Fisher's exact text for unpaired data or a McNemar's test for paired dichotomous data. Measurements taken from the same cell before and after a manipulation, or at multiple time intervals, were compared using a repeated‐measures two‐way analysis of variance (ANOVA) with one factor being the manipulation (or time) and the other being classification by somatic diameter. The data from small and medium–large neurons were pooled if no significant interaction was found between cell size and the manipulation, implying that small and medium–large neurons responded equivalently to the manipulation. *Post hoc* testing was conducted using the Student–Newman–Keuls test. If the underlying distribution was found not to be Gaussian (based on the Lilliefors corrected Kolmogorov–Smirnov test), the Wilcoxon signed‐rank test was used to test the effect of each factor. Independent samples were compared using the Student's *t* test or Mann–Whitney *U* test. Data are reported as the mean ± SEM for normally distributed data, or as the median and interquartile range for non‐normally distributed data. In all studies, statistical significance was defined as *P* < 0.05.

## Results

Unless otherwise stated, we recorded from cutaneous afferents in dissociated DRG cultures identified by retrograde labelling of DiI injected intradermally 10–20 days prior to recording. In one set of experiments, muscle afferents were identified by retrograde labelling of DiI injected intramuscularly and exclusion of Fast Blue injected intradermally. In all cases, soma diameter was used to differentiate small (≤30 μm diameter) and medium–large (>30 μm diameter) neurons, which generally have unmyelinated C fibres and myelinated A fibres, respectively (Harper & Lawson, 1985).

### Baseline spiking characteristics and passive membrane properties

**Figure 2:**
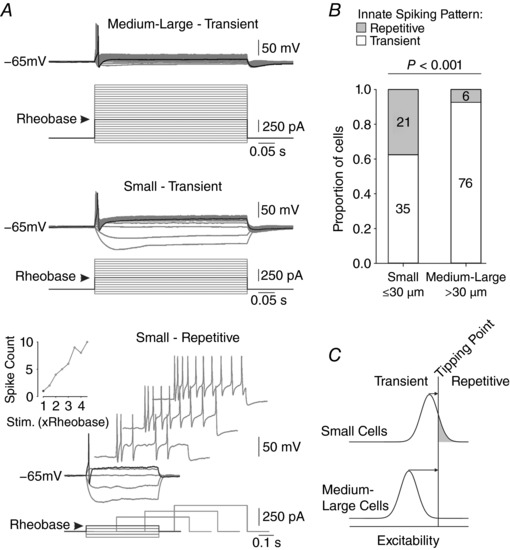
Baseline spiking characteristics of cutaneous somatosensory neurons *A*, sample traces showing typical responses to sustained somatic current injection. Almost all medium–large neurons (top) fire a single spike at stimulus onset once *I*_stim_ reaches rheobase (black trace); this pattern is maintained as *I*_stim_ is increased above rheobase (grey traces). Most small neurons also exhibit transient spiking (middle) but some spike repetitively (bottom) with the spike count increasing as *I*_stim_ is increased above rheobase (inset graph). In all examples, rheobasic stimulation and the corresponding response are shown in black. *B*, summary of the number of neurons exhibiting transient or repetitive spiking. Compared with medium–large neurons, a significantly greater proportion of small neurons exhibited repetitive spiking (χ^2^ = 17.393, *P* < 0.001). *C*, schematic diagram shows how the differential distribution of excitability between small and medium–large neurons could explain the difference in the proportion of spiking patterns reported in *B*. Since some small cells exhibit repetitive spiking at baseline, this population is predicted to operate closer to tipping point than medium–large neurons, few of which spike repetitively at baseline. Operating closer to a tipping point predicts that small cells should be easier to convert to repetitive spiking (i.e. require less ${\bar{g}}_{\mathrm{Na}}$) than medium–large cells.

To determine whether small and medium–large neurons differ in their baseline spiking patterns, the spiking evoked by somatic current injection was compared. Pre‐stimulus *V*_m_ was adjusted to −65 mV in all cells. Two patterns were identified (Fig. 2 *A*): transient and repetitive (see Methods for precise definitions). Figure 2 *B* summarizes the proportion of neurons exhibiting each pattern. Of the 56 small neurons recorded, 35 (63%) displayed transient spiking even to stimulus amplitudes 3–4 times rheobase. Of the 82 medium–large neurons recorded, 76 (93%) were transient spiking. The proportion of repetitive spiking neurons was significantly greater amongst small neurons (χ^2^ = 17.393, *P* < 0.001) (Fig. 2 *B*).

If we assume that excitability (defined here as distance to tipping point) is distributed normally and that the proportion of repetitive spiking neurons represents the part of the distribution located to the right of the tipping point separating the two spiking patterns (Fig. 2 *C*), then the results in Fig. 2 *B* suggest that small neurons, on average, operate closer to their tipping point. This predicts that a smaller perturbation is required to convert small neurons to repetitive spiking than is required to convert medium–large neurons. Only transient spiking neurons were used in subsequent experiments since the goal of our experiments was to convert them to repetitive spiking.

Focusing on transient spiking cutaneous neurons, the passive membrane properties tended to differ between small (*n* = 35) and medium–large (*n* = 76) neurons (Table 1). To account for effects of cell size and isolate true differences in membrane excitability, we normalized certain metrics by membrane capacitance, *C*_m_. After normalizing by *C*_m_, medium–large cells were found to have a significantly higher leak conductance density than small cells (Mann–Whitney *U*_109_ = 568.0, *P* < 0.001), which partly accounts for a significantly higher rheobase/*C*_m_ amongst medium–large cells compared with small cells (Mann–Whitney *U*_109_ = 826.0, *P* < 0.001).

**Table 1:** Properties of transient spiking cutaneous primary somatosensory neurons

	Classification		
	Small	Medium–large	*P*
*n*	35	76	
Resting *V*_m_ (mV)	−65.6 (−72.4 to −61.0)	−72.8 (−76.8 to −68.7)	<0.001
*R*_in_ (MΩ)	618 (341–752)	132 (81–225)	<0.001
τ_m_ (ms)	28.5 (20.9–40.9)	13.1 (7.1–23.1)	<0.001
*C*_m_ (pF)	51.0 (40.2–69.8)	97.6 (69.2–119.1)	<0.001
Rheobase (pA)	250 (105–369)	550 (400–1045)	<0.001
g leak /Cm (nS pF^−1^)	0.035 (0.025–0.048)	0.076 (0.043–0.143)	<0.001
Rheobase/*C*_m_ (pA pF^−1^)	3.9 (2.4–6.1)	5.5 (4.0–11.4)	<0.001

Data show median and the interquartile range. Resting *V*_m_, resting membrane potential; *R*_in_, input resistance; τ_m_, membrane time constant; *C*_m_, membrane capacitance calculated as *C*_m_ = τ_m_/*R*_in_; g leak /Cm, leak conductance density, where g leak =1/R in . *P* values indicate the level of significance of Mann–Whitney *U* tests.

### Converting neuronal spiking pattern from transient to repetitive

**Figure 3:**
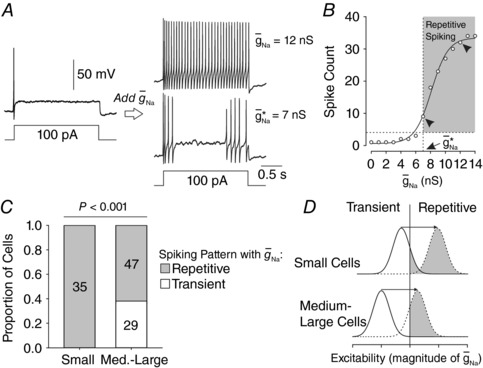
Conversion of spiking pattern using dynamic clamp *A*, sample responses to rheobasic current injection in a typical neuron. The transient spiking observed under control conditions was converted to repetitive spiking when sufficient virtual sodium conductance (${\bar{g}}_{\mathrm{Na}})$ was added using dynamic clamp. By systematically varying ${\bar{g}}_{\mathrm{Na}}$, the minimum conductance required to convert the spiking pattern was identified and is reported as ${\bar{g}}_{\mathrm{Na}}^{*}$. *B*, increasing ${\bar{g}}_{\mathrm{Na}}$ beyond ${\bar{g}}_{\mathrm{Na}}^{*}$ increased the number of spikes evoked by rheobasic stimulation. The horizontal dotted line shows the threshold for repetitive spiking (see Methods). The vertical dotted line highlights the first data point exceeding threshold, and corresponds to ${\bar{g}}_{\mathrm{Na}}^{*}$. *C*, whereas all small neurons could be converted to repetitive spiking by adding enough ${\bar{g}}_{\mathrm{Na}}$, a significant fraction of medium–large neurons were not converted (χ^2^ = 16.155, *P* < 0.001). *D*, schematic diagram shows how the differential rate of spike pattern conversion likely relates to the distance of each cell population from its tipping point, as anticipated in Fig. 2 *C*.

After determining that a neuron spiked transiently to current injection (*I*_stim_) steps, we used dynamic clamp to insert a virtual sodium conductance (see Methods). The virtual conductance resembles Na_v_1.3 channels that are often upregulated after nerve injury (Waxman *et al*. 1994; Kim *et al*. 2001; Fukuoka *et al*. 2008; Huang *et al*. 2008), but the goal of our dynamic clamp manipulation was not to test the effect of sodium channel upregulation *per se* (see Fig. 1 and Discussion). Instead, our strategy was to systematically increase ${\bar{g}}_{\mathrm{Na}}$ while applying *I*_stim_ steps near rheobase to determine the value of ${\bar{g}}_{\mathrm{Na}}$ at which the neuron switched to repetitive spiking (Fig. 3 *A*). Beyond this critical value, denoted ${\bar{g}}_{\mathrm{Na}}^{*}$, increasing ${\bar{g}}_{\mathrm{Na}}$ further increased the number of spikes (Fig. 3 *B*). Figure 3 *C* summarizes the differential conversion rate in small and medium–large neurons. Under baseline conditions, all small neurons were converted to repetitive spiking but 29 of 76 medium–large neurons (38%) could not be converted despite ${\bar{g}}_{\mathrm{Na}}$ being raised as high as possible without destabilizing the recording. The difference in conversion rate between small and medium–large cells was significant (χ^2^ = 16.155, *P* < 0.001) (Fig. 3 *C*), consistent with the prediction in Fig. 2 *C*, namely, that small cells operate closer to their tipping point. In other words, the entire distribution of small cells could be pushed across the tipping point by inserting ${\bar{g}}_{\mathrm{Na}}$ whereas part of the distribution of medium–large cells could not be pushed far enough (Fig. 3 *D*), though we cannot exclude the possibility that some medium–large neurons might never be converted even if larger virtual conductances could be applied.

**Figure 4:**
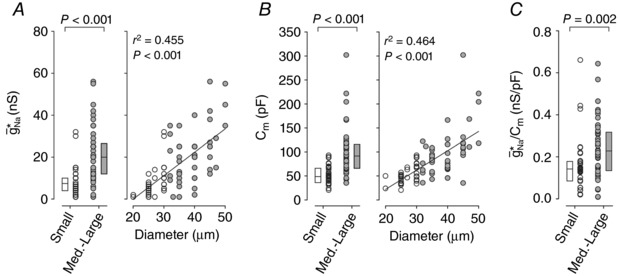
Distance to tipping point differs between small and medium–large neurons *A*, small neurons (white) require more ${\bar{g}}_{\mathrm{Na}}$ to convert their spiking pattern than medium–large neurons (grey). Boxes show the group median and 25th–75th percentiles. The median ${\bar{g}}_{\mathrm{Na}}^{*}$ of 6.0 (4.0–10.0) nS for small neurons was significantly less than the median ${\bar{g}}_{\mathrm{Na}}^{*}$ of 20.0 (12.0–26.5) nS for medium–large neurons (Mann–Whitney *U*_80_ = 276.0, *n*_small_ = 35, *n*_medium–large_ = 47, *P* < 0.001), but this is at least partly due to cell size and may not, therefore, represent a true difference in excitability between putative C‐ and A‐type neurons. Linear regression revealed a significant correlation between soma diameter and ${\bar{g}}_{\mathrm{Na}}^{*}$ (*r* ^2^ = 0.455, *P* < 0.001). *B*, membrane capacitance, *C*_m_, was also correlated with soma diameter (*r* ^2^ = 0.464; *P* < 0.001) and, as expected, differed significantly between small and medium–large neurons (Mann–Whitney *U*_80_ = 276.0, *P* < 0.001). *C*, to isolate true differences in excitability, ${\bar{g}}_{\mathrm{Na}}^{*}$ was normalized by *C*_m_ on a cell‐by‐cell basis, essentially converting the virtual conductance amplitude into a conductance density (${\bar{g}}_{\mathrm{Na}}^{*}$/*C*_m_). The median ${\bar{g}}_{\mathrm{Na}}^{*}$/*C*_m_ of 0.142 (0.085–0.178) nS pF^−1^ in small neurons was significantly less than the median ${\bar{g}}_{\mathrm{Na}}^{*}$/*C*_m_ of 0.228 (0.134–0.319) nS pF^−1^ in medium–large neurons (Mann–Whitney *U*_80_ = 494.0, *P* = 0.002), thus confirming that medium–large neurons do indeed differ in their distance to tipping point.

Values of ${\bar{g}}_{\mathrm{Na}}^{*}$ correlated significantly with soma diameter (*r* ^2^ = 0.455, *P* < 0.001) (Fig. 4 *A*). The median ${\bar{g}}_{\mathrm{Na}}^{*}$ of 6.0 (4.0–10.0) nS in small neurons was significantly less than the median ${\bar{g}}_{\mathrm{Na}}^{*}$ of 20.0 (12.0 – 26.5) nS in medium–large neurons (Mann–Whitney *U*_80_ = 276.0, *P* < 0.001). However, as with certain membrane properties (see Table 1), differences in ${\bar{g}}_{\mathrm{Na}}^{*}$ partly reflect differences in cell size. As expected, *C*_m_ was significantly correlated with soma diameter (*r* ^2^ = 0.464, *P* < 0.001) (Fig. 4 *B*). Thus, to correct for the effect of cell size and thereby isolate differences in excitability, we normalized ${\bar{g}}_{\mathrm{Na}}^{*}$ by *C*_m_. The median ${\bar{g}}_{\mathrm{Na}}^{*}$/*C*_m_ of 0.228 (0.134–0.319) nS pF^−1^ in medium–large neurons was still significantly greater than the median ${\bar{g}}_{\mathrm{Na}}^{*}$/*C*_m_ of 0.142 (0.085–0.178) nS pF^−1^ in small neurons (Mann–Whitney *U*_80_ = 494.0, *P* = 0.002) (Fig. 4 *C*). This difference demonstrates that transient‐spiking small neurons operate significantly closer to their tipping point than medium–large neurons, consistent with the differential distance to tipping point inferred from spiking pattern proportions in Figs 2 *C* and 3 *D*.

### Spiking pattern conversion requirements are stable across time, *V*_m_ and temperature

**Figure 5:**
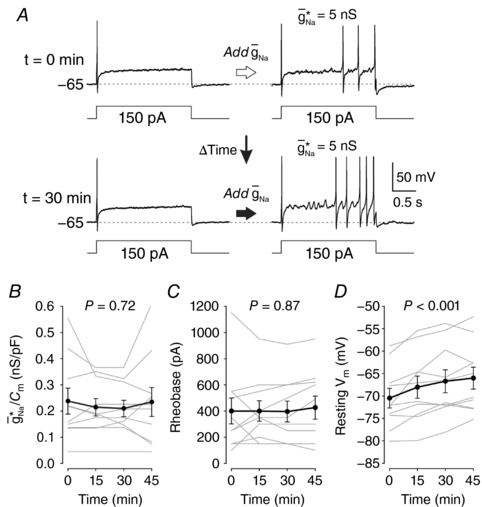
Rheobase and ${\bar{g}}_{\mathrm{Na}}^{*}$ remain stable over the duration of experiments *A*, sample responses from a neuron when first converted to repetitive spiking (top) and again when re‐tested 30 min later (bottom). Rheobase, ${\bar{g}}_{\mathrm{Na}}^{*}$ and resting *V*_m_ were measured at 15 min intervals. Five small cells and 5 medium–large cells were tested but data were pooled across cell types based on the lack of significant interaction between time and cell type (see text). In all subsequent panels, data from individual neurons are shown in grey; group averages (±SEM) are shown in black. *B*, time did not have a significant effect on ${\bar{g}}_{\mathrm{Na}}^{*}$/*C*_m_ (two‐way repeated measures ANOVA, *F*_3,8_ = 0.45, *P* = 0.72). *C*, time did not have a significant effect on rheobase (*F*_3,8_ = 0.24, *P* = 0.87). *D*, time did have a significant effect on resting *V*_m_ (*F*_3,8_ = 12.33, *P* < 0.001). Other measurements were unaffected by this drift since *V*_m_ was maintained at −65 mV except when measuring resting *V*_m_.

A number of control experiments were undertaken to establish the feasibility of subsequent experiments. First, to determine whether ${\bar{g}}_{\mathrm{Na}}^{*}$/*C*_m_ or other cell properties drift over the duration of a typical experiment, we repeated measurements at 15 min intervals in five small cells and five medium–large cells. Data were pooled across cell types based on the lack of interaction between cell size and time on ${\bar{g}}_{\mathrm{Na}}^{*}$/*C*_m_ or rheobase (two‐way repeated measures ANOVAs, *F*_3,8_ = 1.0, *P* = 0.41 and *F*_3,8_ = 0.75, *P* = 0.54, respectively). As illustrated for a typical neuron in Fig. 5 *A*, the innate spiking pattern remained transient over time and was readily switched to repetitive spiking with dynamic clamp. Quantitatively, ${\bar{g}}_{\mathrm{Na}}^{*}$/*C*_m_ did not shift significantly over time (*F*_3,8_ = 0.45, *P* = 0.72) (Fig. 5 *B*) and rheobase was similarly stable (*F*_3,8_ = 0.24, *P* = 0.87) (Fig. 5 *C*). Nor was there any significant shift in *R*_in_ (*F*_3,8_ = 1.18, *P* = 0.34) or τ_m_ (*F*_3,8_ = 0.71, *P* = 0.56). However, there was a significant depolarizing shift in resting *V*_m_ (*F*_3,8_ = 12.33, *P* < 0.001) (Fig. 5 *D*), with the mean (± SEM) voltage shifting from –70.5 ± 2.2 mV at the first test to –66.0 ± 2.4 mV at the fourth test 45 min later. It is, therefore, notable that we standardized the pre‐stimulus *V*_m_ to –65 mV when making all other measurements (see above).

**Figure 6:**
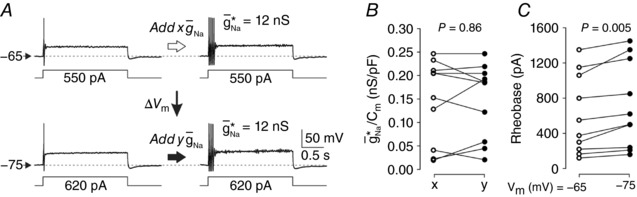
Direct hyperpolarization affects rheobase but not ${\bar{g}}_{\mathrm{Na}}^{*}$ *A*, sample responses from a neuron with *V*_m_ adjusted to –65 mV (top) and again with *V*_m_ reset to –75 mV (bottom). Hyperpolarization caused a predictable increase in rheobase but no change in ${\bar{g}}_{\mathrm{Na}}^{*}$. A change in *V*_m_ is predicted to impact ${\bar{g}}_{\mathrm{Na}}^{*}$ only if channel states are affected (e.g. channels are inactivated) so that the dynamical interaction during rheobasic stimulation is altered; simply requiring more *I*_stim_ to initiate the same dynamics will not impact ${\bar{g}}_{\mathrm{Na}}^{*}$. Five small cells and 5 medium–large cells were tested but data were pooled across cell types based on the lack of significant interaction between membrane potential and cell type (see text). *B*, hyperpolarization did not significantly affect ${\bar{g}}_{\mathrm{Na}}^{*}$/*C*_m_ (two‐way repeated measures ANOVA, *F*_1,8_ = 0.03, *P* = 0.86). *C*, hyperpolarization significantly affected rheobase (*F*_1,8_ = 14.48, *P* = 0.005).

Next, to rule out that changes in ${\bar{g}}_{\mathrm{Na}}^{*}$ always parallel changes in *V*_m_ and rheobase (and thus do not provide independent information), we re‐measured ${\bar{g}}_{\mathrm{Na}}^{*}$ with the pre‐stimulus *V*_m_ adjusted to –65 mV or –75 mV since many studies on afferent hyperexcitability report changes in resting *V*_m_ and rheobase. Though perhaps correlated with a switch in spiking pattern, those changes are neither necessary nor sufficient to explain that switch. Specifically, those metrics do not directly address which non‐linear process will be utilized to generate spikes (Prescott *et al*. 2008) and thus we predicted that a change in *V*_m_ imposed by current injection would not produce a concomitant change in ${\bar{g}}_{\mathrm{Na}}^{*}$, though it would affect rheobase. Testing was conducted on five small cells and five medium–large cells, but results were pooled based on the lack of interaction between cell size and *V*_m_ on ${\bar{g}}_{\mathrm{Na}}^{*}$/*C*_m_ and rheobase (two‐way repeated measures ANOVAs, *F*_1,8_ = 4.76, *P* = 0.061 and *F*_1,8_ = 0.43, *P* = 0.53, respectively). Responses from a typical neuron are illustrated in Fig. 6 *A*. As predicted, hyperpolarization did not significantly affect ${\bar{g}}_{\mathrm{Na}}^{*}$/*C*_m_ (*F*_1,8_ = 0.03, *P* = 0.86) (Fig. 6 *B*) but did yield the expected increase in rheobase (*F*_1,8_ = 14.48, *P* = 0.005) (Fig. 6 *C*).

**Figure 7:**
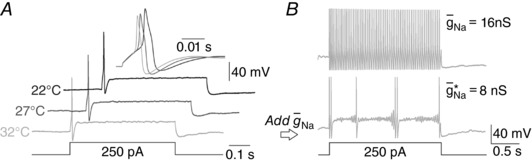
Physiological temperature does not affect spiking pattern or its conversion *A*, sample responses show a consistent spiking pattern as the bath temperature was slowly raised from 22°C to 32°C. Despite no change in rheobase or spiking pattern, the spike amplitude and half‐width were reduced at higher temperatures, consistent with faster channel gating kinetics. *B*, sample traces at 32°C showing the conversion to repetitive spiking by addition of ${\bar{g}}_{\mathrm{Na}}^{*}$ (bottom trace). With *I*_stim_ constant, the spike count increased as ${\bar{g}}_{\mathrm{Na}}$ was increased (top trace), consistent with the pattern observed at room temperature (see Fig. 3 *A* and *B*). The same patterns were observed in 3 of 3 neurons tested.

Lastly, to test whether the innate spiking pattern or our ability to convert it using dynamic clamp was temperature sensitive, experiments were repeated as bath temperature was increased from 22 to 32°C. In 3 of 3 neurons tested, the innate spiking pattern was transient across this temperature range (Fig. 7 *A*) and could be converted to repetitive spiking by introducing *g*_Na_ (Fig. 7 *B*), which demonstrates the feasibility of converting spiking pattern regardless of temperature. We did not compare$\;{\bar{g}}_{\mathrm{Na}}^{*}$ at different temperatures since the kinetics of the virtual conductance should arguably be changed as temperature changes. Indeed, despite the consistency in spiking pattern, individual spikes became shorter and narrower at higher temperature (inset in Fig. 7 *A*). Speeding up the kinetics of the virtual conductance risks destabilizing dynamic clamp recordings and would, therefore, likely reduce the maximal virtual conductance that could be inserted. We did not test whether the effects of other manipulations (e.g. inflammatory mediators) are temperature sensitive, but this could be studied in future experiments.

### Depolarizing shift in *E*_K_ facilitates repetitive spiking in both cell types

The extracellular concentration of potassium ([K*^+^*]_o_) can increase during sustained high‐rate spiking of the cell of interest or its neighbours (Yaari *et al*. 1986) or through leakage of intracellular potassium from damaged cells (Tsantoulas *et al*. 2012), both of which can occur in the context of nerve injury (Amaya *et al*. 2013). Due to the logarithmic relationship between [K^+^]_o_ and the potassium reversal potential (*E*_K_), small changes in [K*^+^*]_o_ can result in relatively large changes in *E*_K_, diminishing the potassium driving force and thereby increasing excitability.

**Figure 8:**
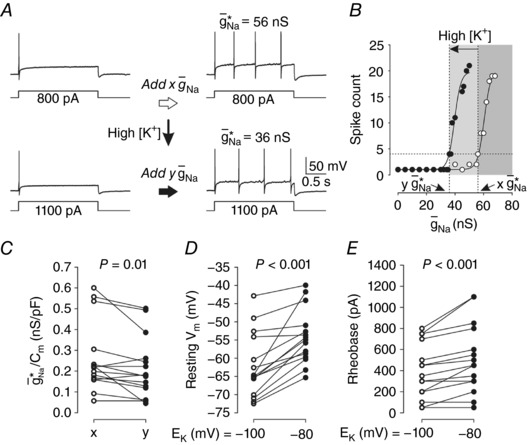
Depolarizing shift in potassium reversal potential (*E*_K_) reduces ${\bar{g}}_{\mathrm{Na}}^{*}$ *A*, sample responses before and after spike pattern conversion by ${\bar{g}}_{\mathrm{Na}}$ under baseline conditions (top) and again after an increase in [K^+^]_o_. Values of ${\bar{g}}_{\mathrm{Na}}^{*}$ before and after the increase in [K^+^]_o_ are denoted *x* and *y*, respectively. The absence of repetitive spiking in response to high [K^+^]_o_ alone (i.e. without dynamic clamp; bottom left trace) was typical. *B*, increasing ${\bar{g}}_{\mathrm{Na}}$ beyond ${\bar{g}}_{\mathrm{Na}}^{*}$ increased the spike count in response to rheobasic *I*_stim_. This relationship was shifted leftward by high [K^+^]_o_. *C*, high [K^+^]_o_ had a significant effect on ${\bar{g}}_{\mathrm{Na}}^{*}$/*C*_m_ (Wilcoxon signed‐rank test, *P* = 0.010). *D*, high [K^+^]_o_ also had a significant effect on resting *V*_m_ (paired *t*_14_ = –5.48, *P* < 0.001). *E*, despite maintaining *V*_m_ at –65 mV for rheobase testing, high [K^+^]_o_ also significantly affected rheobase (Wilcoxon signed‐rank test, *P* < 0.001).

To test how an isolated change in [K*^+^*]_o_ contributes to changes in spiking pattern, we switched the perfusing ACSF from one with 2.5 mm [K*^+^*] to one with 5.5 mm [K*^+^*], which shifts *E*_K_ from –100 to –80 mV according to the Nernst equation. Figure 8 *A* shows sample responses from a typical neuron converted to repetitive spiking in regular ACSF and again after 10 min in ACSF with high [K*^+^*]; ${\bar{g}}_{\mathrm{Na}}^{*}$ in each condition is denoted *x* and *y*, respectively. Increasing ${\bar{g}}_{\mathrm{Na}}$ above ${\bar{g}}_{\mathrm{Na}}^{*}$ increased the number of spikes evoked by rheobasic stimulation, and that relationship was shifted leftward by high [K*^+^*] (Fig. 8 *B*). Based on the 14 neurons tested, high [K*^+^*] significantly reduced ${\bar{g}}_{\mathrm{Na}}^{*}$/*C*_m_ from a median value of 0.218 (0.159–0.363) to 0.178 (0.121–0.257) nS pF^−1^ (Wilcoxon signed‐rank test, *P* = 0.010) (Fig. 8 *C*). The change in ${\bar{g}}_{\mathrm{Na}}^{*}$/*C*_m_ did not differ significantly between small and medium–large neurons (*t*_13_ = –1.18; *P* = 0.26) and data were therefore pooled. Although elevated [K*^+^*]_o_ moved neurons towards their tipping point, this manipulation alone (i.e. without insertion of ${\bar{g}}_{\mathrm{Na}}$) was insufficient to convert the spiking pattern in any neurons tested. This modest effect suggests that a change in [K*^+^*]_o_ is only one of many factors that contribute to causing a switch in spiking pattern in primary somatosensory neurons under pathological conditions.

Effects of high [K^+^]_o_ on resting *V*_m_ and rheobase were controlled for when measuring ${\bar{g}}_{\mathrm{Na}}^{*}$ but, as expected, high [K^+^]_o_ shifted the mean resting *V*_m_ from –71.2 ± 2.3 to –63.2 ± 1.5 mV (paired *t*_14_ = –5.48, *P* < 0.001) (Fig. 8 *D*). In 12 of 15 neurons, high [K^+^]_o_ increased the median rheobase from 350 (200–650) to 450 (300–750) pA (Wilcoxon signed‐rank test, *P* < 0.001) (Fig. 8 *E*). The shift in rheobase is in the opposite direction to that expected, but that is explained by the pre‐stimulus *V*_m_ being held at –65 mV for all rheobase measurements; rheobase would likely have been reduced if the 8 mV shift in resting *V*_m_ had not been controlled for. Neither τ_m_ nor *R*_in_ significantly differed between control and high [K*^+^*]_o_ conditions: The median τ_m_ for baseline and high [K^+^]_o_ groups was 16.6 (9.2–22.9) and 13.8 (10.1–16.4) ms, respectively (Wilcoxon signed‐rank test, *P* = 0.39) and the median *R*_in_ was 318 (84–457) and 159 (91–303) MΩ, respectively (Wilcoxon signed‐rank test, *P* = 0.055).

### Inflammatory mediators facilitate repetitive spiking, especially in medium–large neurons

Inflammatory mediators have been shown to cause repetitive spiking in some somatosensory neurons (see Discussion) but it remains unclear if neurons whose spiking remains transient have nonetheless experienced a subliminal shift toward their tipping point. The implication is that inflammation‐induced facilitation of repetitive spiking will make those neurons more prone to switching spiking pattern if other changes (e.g. high [K*^+^*]_o_) were also to occur. Observation that neurons injured *in vivo* are more prone to switch to repetitive spiking than uninjured neurons when subsequently exposed to inflammatory mediators *in vitro* (Ma *et al*. 2006) supports the notion that repetitive spiking results from the cumulative effect of multiple pathological factors.

**Figure 9:**
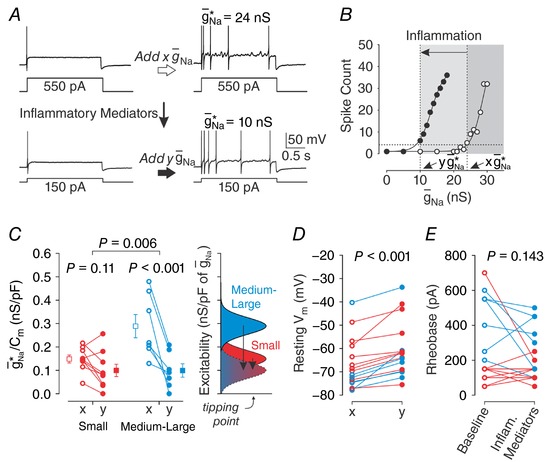
Inflammatory mediators reduce ${\bar{g}}_{\mathrm{Na}}^{*}$ more in medium–large cells than in small cells *A*, sample responses before and after spiking pattern conversion by ${\bar{g}}_{\mathrm{Na}}$ under baseline conditions (top) and again after addition of inflammatory mediators (bottom). Values of ${\bar{g}}_{\mathrm{Na}}^{*}$ before and after addition of inflammatory mediators are denoted *x* and *y*, respectively. The absence of repetitive spiking in response to inflammatory mediators alone (i.e. without dynamic clamp; bottom left trace) was typical. *B*, increasing ${\bar{g}}_{\mathrm{Na}}$ beyond ${\bar{g}}_{\mathrm{Na}}^{*}$ increased the spike count in response to rheobasic *I*_stim_. Inflammatory mediators shifted this relationship leftward. Data from small neurons (*n* = 9, red) and medium–large neurons (*n* = 7, blue) were analysed separately based on the significant interaction between cell size and inflammation (see text). *C*, inflammatory mediators did not have a significant effect on the ${\bar{g}}_{\mathrm{Na}}^{*}$/*C*_m_ of small cells (Student–Newman–Keuls test, *P* = 0.11) but did have a significant effect on medium–large cells (*P* < 0.001). The inflammation‐induced reduction of ${\bar{g}}_{\mathrm{Na}}^{*}$/*C*_m_ was significantly larger in medium–large neurons (*t*_14_ = –3.25, *P* = 0.006). As illustrated by the schematic diagram on the right, and by the group means (±SEM) on the main graph, small and medium–large neurons differed significantly in their ${\bar{g}}_{\mathrm{Na}}^{*}$/*C*_m_ at baseline (Student–Newman–Keuls test, *P* = 0.005) but not after inflammatory mediators (*P* = 0.99). *D*, inflammation had a significant effect on resting *V*_m_ (Wilcoxon signed‐rank test; *P* < 0.001), but that effect did not differ significantly between small and medium–large neurons (Mann–Whitney *U*_14_ = 21.0, *P* = 0.29). *E*, inflammation did not have a significant effect on rheobase (paired *t*_15_ *= *1.54, *P* = 0.14).

To measure the inflammation‐induced shift in the distance to tipping point, we measured ${\bar{g}}_{\mathrm{Na}}^{*}$ before and after adding inflammatory mediators to the ACSF, as illustrated in Fig. 9 *A*. Inflammation shifted the relationship between spike count and ${\bar{g}}_{\mathrm{Na}}$ (Fig. 9 *B*). The effect of inflammation on ${\bar{g}}_{\mathrm{Na}}^{*}$/*C*_m_ was found to depend on cell size as revealed by a significant interaction between these factors (two‐way repeated measures ANOVA, *F*_1,14_ = 10.60, *P* = 0.006), thus data were analysed separately for each cell type. Amongst small cells (*n* = 9), the reduction in mean ${\bar{g}}_{\mathrm{Na}}^{*}$/*C*_m_ from 0.148 ± 0.017 to 0.100 ± 0.027 nS pF^−1^ was not significant (Student–Newman–Keuls test, *P* = 0.11) (Fig. 9 *C* red), whereas amongst medium–large cells (*n* = 7), the reduction in mean ${\bar{g}}_{\mathrm{Na}}^{*}$/*C*_m_ from 0.289 ± 0.051 to 0.099 ± 0.029 nS pF^−1^ was significant (*P* < 0.001) (Fig. 9 *C* blue). The reduction of ${\bar{g}}_{\mathrm{Na}}^{*}$/*C*_m_ was significantly greater in medium–large neurons than in small neurons (*t*_14_ = –3.25, *P* = 0.006). Whereas small and medium–large neurons differed significantly in ${\bar{g}}_{\mathrm{Na}}^{*}$/*C*_m_ at baseline (Student–Newman–Keuls test, *P* = 0.005), they did not differ after exposure to inflammatory mediators (*P* = 0.99). These data argue that medium–large neurons are more strongly affected by inflammatory mediators than are small neurons, but because the latter operate closer to their tipping point under baseline conditions, both cell populations end up equally close to their tipping point after inflammatory mediators are applied. Consistent with this, one cell of each type was converted to repetitive spiking by application of inflammatory mediators alone (without dynamic clamp).

The resting *V*_m_ was significantly depolarized by inflammatory mediators (Wilcoxon signed‐rank test; *P* < 0.001) (Fig. 9 *D*), but that shift did not differ significantly between small and medium–large neurons (Mann–Whitney *U*_14_ = 21.0, *P* = 0.29). Inflammatory mediators did not have a significant effect on rheobase (paired *t*_15_ = 1.54, *P* = 0.14) (Fig. 9 *E*), bearing in mind that changes in resting *V*_m_ were controlled for during rheobase measurements. The effect on rheobase did not differ between small and medium–large neurons (*t*_14_ = 0.79, *P* = 0.44). Inflammatory mediators did not have a significant effect on either τ_m_ (Wilcoxon signed‐rank test, *P* = 0.12) or *R*_in_ (paired *t*_14_ = 0.56, *P* = 0.58).

### Inflammatory mediators also facilitate repetitive spiking in muscle afferents

**Figure 10:**
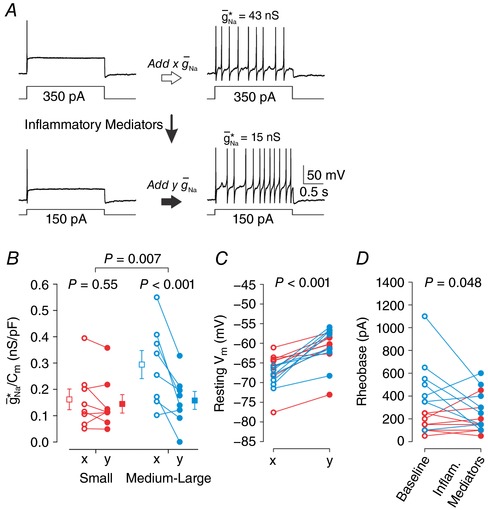
Inflammatory mediators reduce ${\bar{g}}_{\mathrm{Na}}^{*}$ in muscle afferents *A*, sample responses from muscle afferent using same protocol as in Fig. 9 for cutaneous afferents. Data from small neurons (*n* = 8, red) and medium–large neurons (*n* = 8, blue) were analysed separately based on the significant interaction between cell size and inflammation (see text). *B*, inflammatory mediators did not have a significant effect on the ${\bar{g}}_{\mathrm{Na}}^{*}$/*C*_m_ of small cells (Student–Newman–Keuls test, *P* = 0.55) but did have a significant effect on medium–large cells (*P* < 0.001). The inflammation‐induced reduction of ${\bar{g}}_{\mathrm{Na}}^{*}$/*C*_m_ was significantly larger in medium–large neurons (*t*_14_ = –3.19, *P* = 0.007). Like in cutaneous afferents, small and medium–large neurons differed significantly in their ${\bar{g}}_{\mathrm{Na}}^{*}$/*C*_m_ at baseline (Student–Newman–Keuls test, *P* = 0.036) but not after inflammatory mediators (*P* = 0.83). *C*, inflammation had a significant effect on resting *V*_m_ (two‐way repeated measures ANOVA, *F*_1,14_ = 59.09, *P* < 0.001), but the interaction between cell size and inflammation was not quite significant (*F*_1,14_ = 4.05, *P* = 0.064). *D*, there was a significant interaction between cell size and inflammation (*F*_1,14_ = 4.67, *P* = 0.048). Inflammation significantly reduced rheobase in medium–large cells (Student–Newman–Keuls test, *P* = 0.02) but not in small cells (*P* = 0.67).

Past studies have reported that muscle afferents are more likely than cutaneous afferents to spike spontaneously (Michaelis *et al*. 2000) or to respond to sustained depolarization with repetitive spiking (Liu *et al*. 2002; Ratté *et al*. 2014) after nerve injury. Therefore, to compare with cutaneous afferents, we used the same approach described above to test the effects of inflammatory mediators on muscle afferent excitability. Figure 10 *A* shows responses from a typical neuron. Like in cutaneous afferents, the effect of inflammation on ${\bar{g}}_{\mathrm{Na}}^{*}$/*C*_m_ was found to depend on cell size (two‐way repeated measures ANOVA, *F*_1,14_ = 10.18, *P* = 0.007) and data were thus analysed separately for each cell type. Amongst small cells (*n* = 18), the reduction in mean ${\bar{g}}_{\mathrm{Na}}^{*}$/*C*_m_ from 0.162 ± 0.039 to 0.145 ± 0.035 nS pF^−1^ was not significant (Student–Newman–Keuls test, *P* = 0.55) (Fig. 10 *B* red), whereas amongst medium–large cells (*n* = 18), the reduction in mean ${\bar{g}}_{\mathrm{Na}}^{*}$/*C*_m_ from 0.294 ± 0.053 to 0.158 ± 0.034 nS pF^−1^ was significant (Student–Newman–Keuls test, *P* < 0.001) (Fig. 10 *B* blue). The reduction of ${\bar{g}}_{\mathrm{Na}}^{*}$/*C*_m_ was significantly greater in medium–large neurons than in small neurons (*t*_14_ = –3.19, *P* = 0.007). Whereas small and medium–large neurons differed significantly in ${\bar{g}}_{\mathrm{Na}}^{*}$/*C*_m_ at baseline (Student–Newman–Keuls test, *P* = 0.036), they did not differ after exposure to inflammatory mediators (*P* = 10.83).

Resting *V*_m_ was significantly depolarized by inflammatory mediators (two‐way repeated measures ANOVA, *F*_1,14_ = 59.09, *P* < 0.001) (Fig. 10 *C*). This was true in both small and medium–large neurons (Student–Newman–Keuls test, *P* < 0.001 for each cell type) and the interaction between cell size and inflammatory mediators was not significant (*F*_1,14_ = 4.05, *P* = 0.064). With respect to rheobase, there was a significant interaction between cell size and inflammatory mediators (two‐way repeated measures ANOVA, *F*_1,14_ = 4.67, *P* = 0.048) (Fig. 10 *D*) with medium–large cells experiencing a significant decrease (Student–Newman–Keuls test, *P* = 0.02) whereas small cells experienced no change (*P* = 0.67). Inflammatory mediators did not have a significant effect on either τ_m_ (paired *t*_14_ = –0.51, *P* = 0.62) or *R*_in_ (Wilcoxon signed‐rank test, *P* = 0.40).

**Figure 11:**
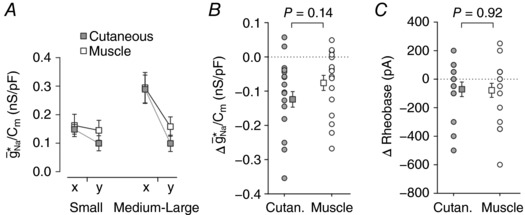
Cutaneous and muscle afferents respond similarly to inflammatory mediators *A*, group means (±SEM) of ${\bar{g}}_{\mathrm{Na}}^{*}$ before (*x*) and after (*y*) inflammation in cutaneous (grey) and muscle afferents (white) collated from Figs 9 and 10, respectively. *B*, the inflammation‐induced reduction in ${\bar{g}}_{\mathrm{Na}}^{*}$/*C*_m_ (measured as *x *−* y*) did not differ significantly between cutaneous and muscle afferents (two‐way ANOVA, *F*_1,31_ = 2.26, *P* = 0.14), nor was there any significant interaction between cell size and the tissue innervated (*F*_1,31_ = 0.05, *P* = 0.82). *C*, the inflammation‐induced reduction in rheobase did not differ significantly between cutaneous and muscle afferents (*F*_1,31_ = 0.01, *P* = 0.92), nor was there any significant interaction between cell size and the tissue innervated (*F*_1,31_ = 2.56, *P* = 0.12).

The pattern of inflammation‐induced changes was similar between cutaneous and muscle afferents. Figure 11 *A* reports the average ${\bar{g}}_{\mathrm{Na}}^{*}$/*C*_m_ before and after inflammatory mediators for each group of cells, collating the group averages shown in Fig. 9 *C* and 10 *B*. To compare the effects of inflammatory mediators, we calculated the inflammation‐induced reduction in ${\bar{g}}_{\mathrm{Na}}^{*}$/*C*_m_ as *x* − *y*. The change in ${\bar{g}}_{\mathrm{Na}}^{*}$/*C*_m_ did not differ significantly between cutaneous and muscle afferents (two‐way ANOVA, *F*_1,31_ = 2.31, *P* = 0.14) (Fig. 11 *B*), nor was there any interaction between cell size and the tissue innervated (*F*_1,31_ = 0.14, *P* = 0.71). The same analysis conducted on the inflammation‐induced shift in rheobase likewise revealed no significant difference between cutaneous and muscle afferents (*F*_1,31_ = 0.00; *P* = 0.98; Fig. 11 *C*) or any interaction between cell size and the tissue innervated (*F*_1,31_ = 1.07; *P* = 0.31). Lastly, the inflammation‐induced shift in resting *V*_m_ did not differ between cutaneous and muscle afferents (Mann–Whitney *U*_30_ = 128, *P* = 0.99). Overall, these results argue that cutaneous and muscle afferents respond similarly to the cocktail of inflammatory mediators that we tested, which contrasts the differential effect of inflammation on small *vs*. medium–large neurons (see Figs 9 and 10). Our results do not contradict past studies reporting differences between cutaneous and muscle afferents but, rather, argue that those differences are due to other inflammatory mediators or to other aspects of nerve injury.

### Variation in repetitive spiking pattern revealed by dynamic clamp

The switch to repetitive spiking reflects a switch in the underlying spike initiation mechanism such that rapid depolarization (driven by an external stimulus) becomes unnecessary for positive feedback activation of fast sodium channels to outcompete slower processes such as delayed‐rectifier potassium channel activation (Prescott *et al*. 2008; Ratté *et al*. 2015). This means that sustained depolarization, whether caused by stimulation or by factors affecting resting *V*_m_, is sufficient to drive spiking, which is of course important for spontaneous spiking, where there is no external input causing rapid depolarization. Hyperexcitable neurons can fire spontaneously in bursts or in a regular pattern (Song *et al*. 1999; Zhang *et al*. 1999; Liu *et al*. 2002; Ma *et al*. 2003). We have shown previously that adaptive processes that are slow relative to spike kinetics are essential for bursting (Rho & Prescott, 2012). This raises the question of whether neurons have latent differences in their adaptation that only become evident when the neuron becomes capable of repetitive spiking, or whether adaptation is also pathologically altered. The two possibilities are not mutually exclusive, but the former possibility is typically overlooked.

**Figure 12:**
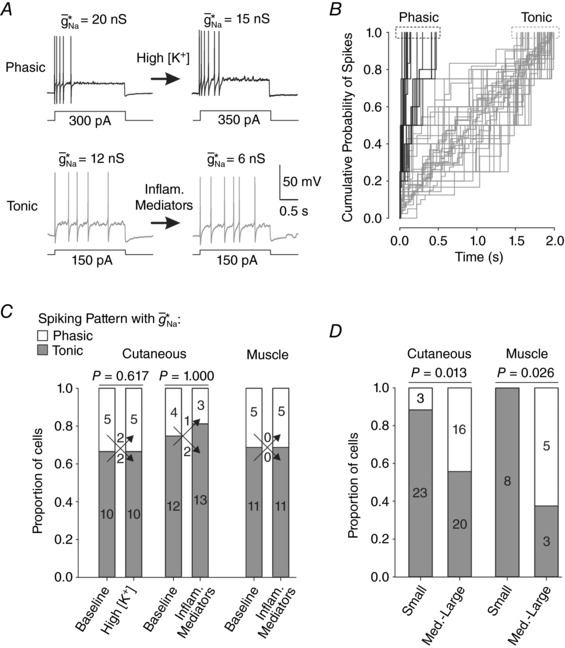
Spike pattern conversion reveals latent differences between cell types *A*, sample traces on left show repetitive spiking during rheobasic stimulation in two neurons after inserting ${\bar{g}}_{\mathrm{Na}}^{*}$ before (left) and during (right) elevation of [K^+^]_o_ (top) or application of inflammatory mediators (bottom). Two subtypes of repetitive spiking were observed: phasic (black), in which spiking abruptly ceases before the end of *I*_stim_ steps due to strong adaptation, and tonic (grey), in which spiking continues throughout *I*_stim_ steps because adaptation is weaker. Sample traces on the right suggest that the pattern of repetitive spiking is independent of the pathomimetic manipulations. As *I*_stim_ is increased above rheobase or as ${\bar{g}}_{\mathrm{Na}}$ is increased above ${\bar{g}}_{\mathrm{Na}}^{*}$, phasic spiking may transition to tonic spiking (data not shown). In the subsequent analysis, we focus on the repetitive spiking pattern at the tipping point, namely, with rheobasic *I*_stim_ and ${\bar{g}}_{\mathrm{Na}}^{*}$. *B*, using spike times from the response to 2 s‐long rheobasic *I*_stim_ and ${\bar{g}}_{\mathrm{Na}}^{*}$, the cumulative probability of spiking plots revealed two profiles that correspond to phasic (black) and tonic (grey). *C*, proportions of neurons exhibiting tonic and phasic spiking under baseline conditions and during high [K^+^] or inflammatory mediators. Amongst cutaneous afferents, the number of neurons switching between patterns (indicated by arrows) was not significant for high [K^+^] (McNemar's test, χ^2^ = 0.250; *P* = 0.617) or inflammatory mediators (χ^2^ = 0.000; *P* = 1.000). All muscle afferents exhibited the same pattern before and after inflammatory mediators. These data suggest that the pattern of repetitive spiking is independent of the manipulations used to facilitate repetitive spiking. *D*, proportion of phasic spiking was significantly greater amongst medium–large neurons than amongst small neurons for cutaneous afferents (χ^2^ = 6.221, *P* = 0.013) and muscle afferents (Fisher's exact test, *P* = 0.026). These data suggest that the pattern of repetitive spiking is dependent on the cell type.

By adding virtual *g*_Na_ to cause repetitive spiking, we observed latent differences in the pattern of repetitive spiking at the tipping point. Figure 12 *A* shows typical examples of phasic (top) and tonic (bottom) patterns of repetitive spiking. Phasic spiking was defined as repetitive spiking that stopped before the end of the *I*_stim_ step (consistent with strong adaptation) whereas tonic spiking was defined as repetitive spiking that continued throughout the *I*_stim_ step (consistent with weak adaptation). Based on the response during dynamic clamp insertion of ${\bar{g}}_{\mathrm{Na}}^{*}$, the cumulative probability of spiking during rheobasic *I*_stim_ revealed two recognizable clusters (Fig. 12 *B*).

Next, we asked whether the pattern of repetitive spiking depended on the manipulation used to cause repetitive spiking (i.e. dynamic clamp alone *vs*. dynamic clamp plus high [K^+^]_o_ or inflammatory mediators). Consistent with examples in Fig. 12 *A*, where each row represents a different neuron, analysis in Fig. 12 *C* argues that the pattern of repetitive spiking is independent of the manipulation. Specifically, of the 15 cutaneous afferents converted to repetitive spiking before and after application of high [K^+^]_o_, two switched from phasic to tonic spiking in high [K^+^]_o_, and another two switched in the opposite direction, which does not represent a significant change in repetitive spiking pattern (McNemar's test; χ^2^ = 0.250; *P* = 0.617). Of the 16 cutaneous afferents converted to repetitive spiking before and after application of inflammatory mediators, two switched from phasic to tonic spiking in inflammatory mediators, and one switched in the other direction, which is also not significant (χ^2^ = 0.000; *P* = 1.000). Of the 16 muscle afferents converted to repetitive spiking, all of them exhibited the same pattern of repetitive spiking before and after application of inflammatory mediators. These results suggest that the pattern of repetitive spiking does not depend on the manipulations, though we cannot exclude that other manipulations that facilitate repetitive spiking – especially those that do so without any virtual sodium conductance being added – might facilitate a certain pattern of repetitive spiking.

Consistent with the latent pattern of repetitive spiking being an intrinsic neuronal property, the proportion of phasic spiking was significantly more common amongst medium–large neurons than amongst small neurons (*χ^2^* = 6.221, *P* = 0.013 for cutaneous afferents; Fisher's exact test, *P* = 0.026 for muscle afferents) (Fig. 12 *D*). This difference in repetitive spiking pattern is consistent with differences in slow adaptive processes, the implication being that different cell types are predisposed to different patterns of pathological spiking not necessarily because they experience different pathological changes (e.g. slow adaptive processes are compromised in one cell type but not another) but, rather, because the same pathological change (e.g. a switch to repetitive spiking) manifests differently because of pre‐existing differences between cell types.

## Discussion

In this study, we have demonstrated a novel way in which dynamic clamp can be used to explore pathological changes in spiking pattern. Primary somatosensory neurons normally respond to abrupt somatic depolarization with transient spiking at the onset of stimulation (see Fig. 2), but, under conditions associated with chronic pain, some neurons develop the capacity to spike repetitively during sustained depolarization (Liu *et al*. 2000, 2002; Xing *et al*. 2001; Ma & LaMotte, 2007; Fan *et al*. 2011; Xie *et al*. 2011; Song *et al*. 2012). This switch in spiking pattern, whether assessed by stimulation or inferred from spontaneous spiking, occurs in only a fraction of neurons and is more common in muscle afferents than in cutaneous afferents (Michaelis *et al*. 2000; Liu *et al*. 2002; Ratté *et al*. 2014), but grossly altered spiking in even a few neurons may have major sensory consequences. Moreover, our data suggest that most (if not all) “unaffected” neurons experience a subliminal change in excitability that makes them more prone to switch their spiking pattern if additional insults were to be experienced by that neuron.

By inserting a virtual sodium conductance known to facilitate repetitive spiking, and measuring exactly how much of that conductance is required to cause repetitive spiking – the liminal conductance change that we refer to as ${\bar{g}}_{\mathrm{Na}}^{*}$ (see Fig. 1) – we showed that small neurons are more prone to repetitive spiking than are medium–large neurons under control conditions. Small and medium–large neurons typically have unmyelinated or myelinated fibres, respectively. By combining dynamic clamp with other pathomimetic manipulations, namely high [K^+^]_o_ and inflammatory mediators, we also showed how the effect of these pathological factors on excitability can be indirectly measured based on how much they reduce ${\bar{g}}_{\mathrm{Na}}^{*}$. Using this approach, we found that high [K^+^]_o_ had similar effects on the excitability of small and medium–large neurons whereas inflammatory mediators had a significantly greater effect in medium–large neurons.

### Different ways of using dynamic clamp

Dynamic clamp was developed over two decades ago (Robinson & Kawai, 1993; Sharp *et al*. 1993) but remains relatively uncommon, especially in studies examining pathophysiological changes associated with pain. Dynamic clamp has recently been applied to primary somatosensory neurons to explore the effects of specific changes in sodium channels (Vasylyev *et al*. 2014; Han *et al*. 2015; Estacion & Waxman, 2017), potassium channels (Ritter *et al*. 2015) and GABA_A_ receptor signalling (Takkala *et al*. 2016). Beyond testing the effects of known molecular changes, our group has used dynamic clamp to reproduce known changes in cellular excitability in order to explore the underlying molecular changes in a more general way, including how such changes may combine to affect excitability (Ratté *et al*. 2014). The virtual sodium conductance that we inserted in this study mimics Na_v_1.3 channels that can be upregulated after nerve injury (Waxman *et al*. 1994; Kim *et al*. 2001; Fukuoka *et al*. 2008; Huang *et al*. 2008), but even if that particular molecular change is not induced in a certain cell by a certain injury, the manipulation nonetheless enables one to study how a neuron operates under two well‐defined conditions (i.e. with and without the added conductance). In other words, the added conductance serves as an acute and precisely controllable perturbation to explore excitability (Tomaiuolo *et al*. 2012; Zeberg *et al*. 2015). By titrating the virtual conductance, we determined how strong the perturbation must be to cause a qualitative change in spiking pattern. We introduced this “distance‐to‐tipping‐point” measurement previously (Ratté *et al*. 2014) but demonstrate here how it can be adapted to measure *modulation* of the distance to tipping point.

Before discussing modulation of the distance to tipping point, it is worth comparing distance‐to‐tipping‐point measurements across different types of neurons as those data provide significant insight into the susceptibility of neurons to switch to repetitive spiking. Indeed, an understanding of that susceptibility and how different molecular changes affect excitability are both necessary for a more holistic understanding of how neuronal excitability becomes pathologically altered.

### Differential susceptibility to spiking pattern changes

Most of the neurons that we tested spiked transiently under baseline conditions but a significantly higher proportion of small cells than medium–large cells exhibited repetitive spiking (Fig. 2). This observation suggests that small cells, on average, operate closer to their tipping point than do medium–large cells. Other data corroborated that interpretation: a higher proportion of small cells could be converted to repetitive spiking by dynamic clamp (Fig. 3) and significantly less virtual conductance was required for that conversion even after accounting for differences in cell surface area (Fig. 4). As a technical note, most neurons that we tested could be converted from transient to repetitive spiking once sufficient virtual sodium conductance was added; those that were not converted may reflect either a technical limitation on the maximum current we could apply without destabilizing the recording, though we cannot exclude the possibility that some medium–large neurons might never be converted, even if very large virtual conductances could be applied. Compared with cutaneous afferents, muscle afferents appeared to operate at a similar distance from their tipping point (Fig. 11) though neurons innervating other tissues (e.g. joints, or viscera) may differ in this regard.

If different cells operate at different distances from their tipping point, they will require more or less *net* molecular change – sodium channel upregulation, potassium channel downregulation, or myriad other changes and any combination thereof – to convert their spiking pattern. Under these conditions, the magnitude of the net molecular change is not sufficient to predict whether spiking pattern will be switched as this also depends on the susceptibility of the neuron. Our data suggest that small cells (with unmyelinated fibres) are switched to repetitive spiking by molecular changes that are too subtle to affect the spiking pattern of medium–large cells (with myelinated fibres). Yet the impact of molecular changes also differs between cell types (see below). The similarity between small and medium–large cells suggested by their transient spiking pattern may belie differences in the underlying balance of different ion channels that can manifest as different patterns of repetitive spiking (Fig. 12) and differential susceptibility to perturbation, reminiscent of recent studies in other systems (Rinberg *et al*. 2013; Sakurai *et al*. 2014). We are unaware of any comparable studies directly addressing this issue in somatosensory afferents.

### Measuring modulation of the distance to tipping point

Beyond measuring the distance to tipping point under a single condition, we have introduced in this study how to measure the contribution of individual pathophysiological changes based on how they modulate the distance to tipping point. Specifically, by measuring (1) how much virtual sodium conductance is required to convert the spiking pattern of a given neuron under baseline conditions, and (2) how much less of that conductance is required when applied in conjunction with another pathomimetic manipulation, the contribution of the second manipulation can be measured indirectly (see Fig. 1). Using this approach, we have shown that high [K^+^]_o_ facilitates repetitive spiking to the same degree in small and medium–large neurons (Fig. 8) whereas inflammatory mediators facilitate repetitive spiking more in medium–large neurons (Figs 9 and 10).

As alluded to above, the magnitude of excitability changes must be considered in light of how near or far different cell types operate from their tipping point. Although inflammation shifted medium–large neurons more towards their tipping point than it did for small neurons, the medium–large neurons were not more likely to convert to repetitive spiking than small neurons because the former normally operate further from their tipping point (see above). More generally, both high [K^+^]_o_ and inflammation pushed neurons towards their tipping point, but neither, on its own (i.e. without dynamic clamp), caused repetitive spiking except in a very small minority of neurons. This is consistent with small effects from multiple different pathological factors needing to summate to cause gross changes in spiking. It is possible that some pathological changes may also interact non‐linearly (i.e. cooperate or interfere with each other) rather than summing linearly. In any case, only a subset of neurons (i.e. those in the tail of the Gaussian distribution describing excitability) are likely to experience a change in spiking pattern unless the net molecular change is especially large. Consistent with this, Ma *et al*. (2006) showed that chronic compression of the dorsal root ganglion caused spontaneous spiking in ∼10% of neurons, but inflammatory mediators applied to injured neurons more than doubled that percentage (with equal effects observed in small, medium and large neurons); the same inflammatory mediators applied to uninjured neurons caused spontaneous spiking in only ∼1% of all neurons. Song *et al*. (2003) reported similar findings but, in contrast, Gold *et al*. (1996) reported that PGE_2_ sensitized 56% of neurons harvested from uninjured animals. In the latter study, sensitization was defined as increased spiking and/*or* reduced spike threshold, which makes it unclear what fraction of neurons switched spiking pattern, and the reported 300% increase in spike number is based only on “sensitized” neurons. On the one hand, this means that at least 44% of neurons exhibited no increase in spike number, let alone tripling, but, on the other hand, one would be remiss in assuming 44% of neurons were unaffected insofar as they may have been shifted toward their tipping point. Notably, we tested acute application of only a few inflammatory mediators; chronic exposure and/or other mediators may cause repetitive spiking (i.e. have a supraliminal effect) in a larger fraction of neurons.

In summary, this study presents a novel approach to quantify how different pathological factors contribute to qualitative changes in neuronal excitability, and whether different cell types are equally prone to a switch in spiking pattern. Our approach, which involves applying an artificial, yet quantifiable, perturbation to a neuron and assessing how that neuron responds, is conceptually distinct from the more traditional approach of measuring how nerve injury or inflammation affects different ion channels and correlating those molecular changes with changes in neuronal excitability. Necessity‐testing experiments in which a particular ion channel is blocked or knocked down/out are invaluable in helping to establish causal links, but quantifying the manipulation tends to be difficult; for example, conducting the required recordings in the same neuron before and after blocking a channel of interest may be technically impossible if certain other channels must be blocked in voltage clamp but not in current clamp. Our dynamic clamp experiments constitute sufficiency‐testing and, as demonstrated, can be very quantitative. Beyond adding (or subtracting) specific conductances to test their direct effects on excitability, we have shown here how titrating the added conductance can be used to gauge the susceptibility of a neuron to switch its spiking pattern, and whether pathological factors like inflammation predispose a neuron to switching. This is particularly valuable for deciphering if and how the effects of co‐occurring factors combine to cause gross changes in neuronal excitability.

## Additional information

### Competing interests

None declared.

### Author contributions

Experiments were performed in the laboratory of S.A.P. (Neurosciences and Mental Health, The Hospital for Sick Children). P.T. and S.A.P. conceived the design of the study. P.T. acquired the data. P.T. and S.A.P. analysed and interpreted the data. P.T. and S.A.P. wrote the manuscript, and revised it critically for important intellectual content. S.A.P. supervised the project. Both authors have read and approved the final version of the manuscript, and agree to be accountable for all aspects of the work in ensuring that questions related to the accuracy or integrity of any part of the work are appropriately investigated and resolved. All persons designated as authors qualify for authorship, and all persons qualified for authorship are listed as authors.

### Funding

This study was supported by grants from the Canadian Institutes of Health Research (CIHR PJT‐153183) and the Natural Sciences and Engineering Research Council of Canada (NSERC RGPIN 436168) to S.A.P. S.A.P. is also a Canadian Institutes of Health New Investigator and recipient of an Ontario Early Researcher Award. P.T. was supported by Ontario Graduate Scholarships (OGS), the SickKids Research Institute and awards from the Institute of Medical Science and the Ontario Student Opportunity Trust Funds (OSOTF).
